# Extraction, Identification, Modification, and Antibacterial Activity of Histone from Immature Testis of *Atlantic salmon*

**DOI:** 10.3390/md18030133

**Published:** 2020-02-26

**Authors:** Boyu Fu, Hong Lin, Tushar Ramesh Pavase, Nasha Mi, Jianxin Sui

**Affiliations:** College of Food Science and Engineering, Ocean University of China, Qingdao, Shandong 266003, China; 13833210033@163.com (B.F.); linhong@ouc.edu.cn (H.L.); tusharpavase@yahoo.com (T.R.P.); minasha@ouc.edu.cn (N.M.)

**Keywords:** Atlantic salmon *(Salmo salar*), histone, extraction, antibacterial activity, enzymatic hydrolysis

## Abstract

In the present study, histone from immature testis of Atlantic salmon was extracted and identified, and its antibacterial activity after enzymolysis was investigated. Histone extracted from Atlantic salmon *(Salmo salar*) testis using the acid extraction method was successfully identified by LC-MS/MS, and revealed significant inhibitory activity on both the Gram-negative and Gram-positive bacteria. With a low concentration of 10 mg/mL, the observed inhibitory zone diameter (IZD) could significantly reach up to 15.23 mm. After modification of enzymatic hydrolysis by pepsin, histone could be digested to three fragments, while the antibacterial activity increased up to 57.7%. All the results suggested the leftovers from commercial fishing could be utilized for the extraction of antimicrobial peptides.

## 1. Introduction

Antimicrobial peptides (AMPs) are small molecular peptides with a varying number of amino acids known to have a broad-spectrum antimicrobial activity [1,2]. Most AMPs, generally consisting of 12–100 amino acid residues, are amphiphilic cationic peptides containing hydrophobic amino acids [3]. AMPs have been recognized as one of the most efficient alternatives to the existing use of antibiotics [4], revealing its major role in innate immunity characterized by broad-spectrum, high efficiency, selective toxicity, stability, and low drug resistance, respectively [5].

Protamine, which is a polycationic peptide existing in the mature testis of fish with a molecular weight of 4.0–4.5 KDa [6,7], is known to possess a variety of biological activities such as bacterial inhibition, anticoagulation, and promoting digestion [8,9,10]. In a previous report, histone has been determined as a nucleoprotein that tightly bounds to the DNA [11]. Meanwhile, the large amounts of lysine content in histones have led to it emerging as a potential food preservative, mainly owing to its stable structure and broad-spectrum antibacterial activity.

Several previous studies have reported that histones have significant inhibitory activity on aquatic organisms. Fernandes et al. (2004) reported that oncorhyncin II, which was a histone-derived peptide, isolated and purified from the acidic extract of skin secretion of rainbow trout (*Oncorhynchus mykiss*), revealed significant antimicrobial activity against both Gram-positive (G+) and Gram-negative (G−) bacteria [12]. The acidic extract from the epidermal mucus of the Atlantic cod (*Gadus morhua*) showed antimicrobial activity against *Bacillus megaterium*, *Escherichia coli*, and *Candida albicans* [13]. De Zoysa et al. (2009) discovered a highly efficient histone-derived antimicrobial peptide from *Haliotis discus discus*, which could be induced in blood cells, digestive tract, and colony tissues after infecting Abalone with various bacteria **[14]**. Histone had also revealed high inhibitory activity on human monocytic leukemia cancer cells (THP-1) in vitro, while the survival rate of normal cells in the control group did not decline [15]. Eskandarian et al. (2013) discovered that histone modification was an important epigenetic modification, which precisely regulated the innate immune response and the expression of corresponding defense genes [16].

The previous studies on AMPs extracted from the testis of Atlantic salmon were mainly focused on the extraction of protamine and its antibacterial activity, with very little information available on the application of histone in immature testis of Atlantic salmon [17]. Studies on the maturation stage of fish sperm cells have revealed that chromatin in the inactive state is composed of histone polymers encased in DNA to form nucleosomes, which are then folded into higher-order structures by nucleosome fibers. As the testes mature, protamine replaces histones and binds to DNA to form highly dense sperm chromatin [18]. The composition and content of nuclein in the testis of fishes with different maturity were different. There was almost no protamine in immature fish testicles, and the amount of protamine increased during the maturation of testis, reaching a large amount at the end of maturation when protamine bound to DNA as a substitute histone for nucleosin [19]. Therefore, mature fish sperms were more suitable for protamine extraction, while the immature fish sperms comprised of a fairly large amount of histone. Owing to the advanced commercial fishing technology and the increase in overfishing, a large proportion of *Atlantic salmon* males was caught before the maturation of their gonads and maturity stage. In addition, the gonads of *Atlantic salmon* males were often treated as leftovers for fodder, fertilizer, or abandoned, resulting in a great amount of waste. Therefore, in this study, the histone was extracted and identified from immature testis of *Atlantic salmon* and its antibacterial activity before and after modification was mainly investigated to obtain the best natural antimicrobial peptide fragments.

## 2. Results

### 2.1. SDS-Page and Tris-Tricine-Sds-Page Electrophoresis

**Figure 1:**
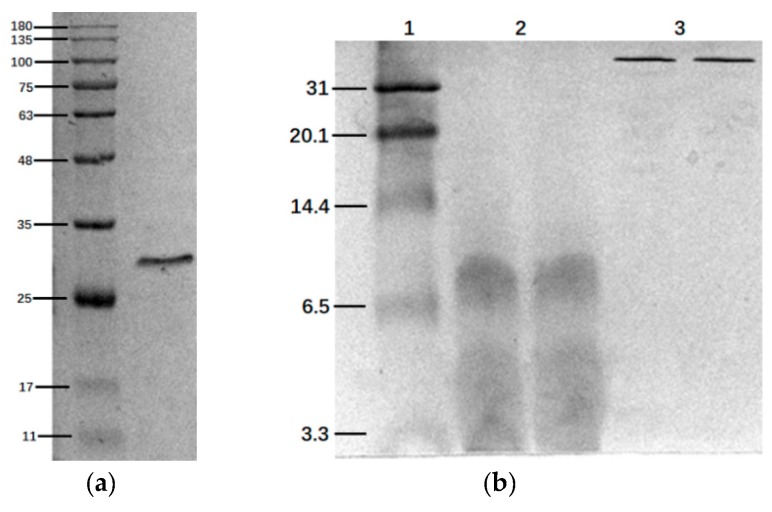
The results of SDS-page electrophoresis and Tris-Tricine-SDS-PAGE. 1: Marker. 2: Commercial protamine. 3: Histone. (**a**) The SDS-page of histone; (**b**) the Tris-Tricine-SDS-PAGE of sigma’s commercial protamine and histone.

The histone extracted from salmon sperm was a single component with the relative molecular weight of about 30 KDa (Figure 1a); it was much bigger than that of the commercial protamine, which was known to have two bands with the molecular weight of 4 KDa and 7 KDa (Figure 1b), respectively.

### 2.2. Histone Identification 

**Figure 2:**
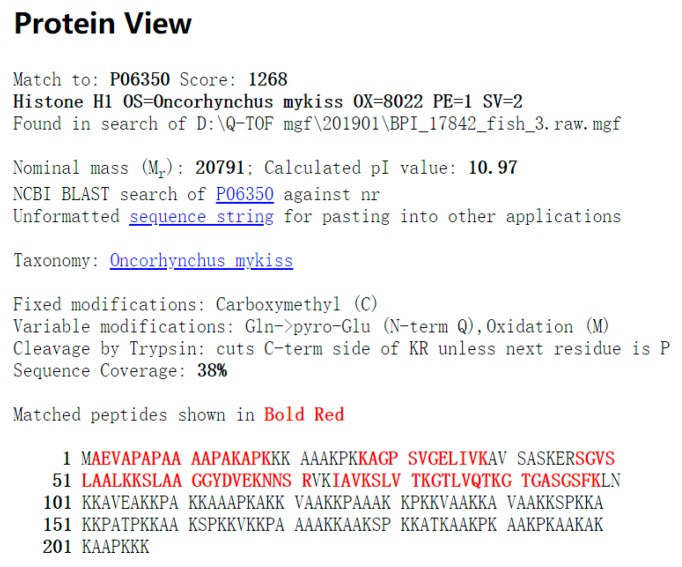
The result of Mascot search in Uniprot. It showed the highest score of protein in salmon histone.

The extracted histone was identified by LC-MS/MS and the peak was labeled using data analysis software, after which the Mascot search in Uniprot was performed. The search conditions and parameters are shown in Table 1, and the partial comparison results in the Uniprot database are shown in Table 2. The results revealed that the highest matching degree of the extracted protein was salmon histone H1, with 38% matching peptide coverage and 1268 evaluation scores (Figure 2). Although the matching degree was not so high, it could be confirmed that the extracted product was histone according to its specific fragment and fraction judgment. There was a significant difference between the above 27 scores, which was an important result obtained.

### 2.3. Histone Modification

**Figure 3:**
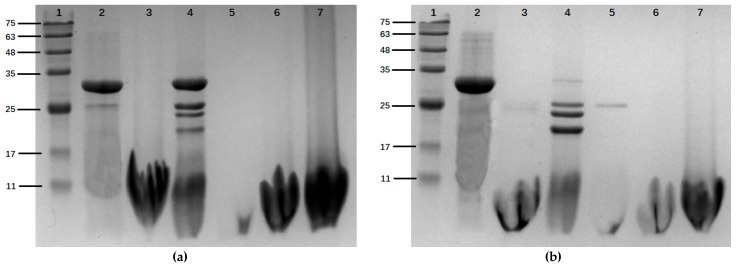
The results of SDS-page electrophoresis of histone after enzymatic hydrolysis. 1: Marker. 2: Histone. 3: Histone treated by papain. 4: Histone treated by pepsin. 5: Histone treated by trypsin. 6: Histone treated by flavor protease. 7: Histone treated by alkaline protease. (**a**) The results of an hour’s hydrolysis of five enzymes; (**b**) the results of five hour’s hydrolysis of five enzymes.

Histone was modified by protease hydrolysis with papain, pepsin, trypsin, flavor protease, and alkaline protease. When incubated for 1 h, histone was completely hydrolyzed by papain, trypsin, flavor protease, and alkaline protease, and three other bands appeared after pepsin hydrolysis (Figure 3a). After 5 h incubation, histone was completely hydrolyzed by pepsin, which was observed by the three new bands at molecular weights of about 25, 23, and 19 KDa, respectively (Figure 3b). 

### 2.4. Antibacterial Activity Results

#### 2.4.1. Histone Antimicrobial Activity

**Figure 4:**
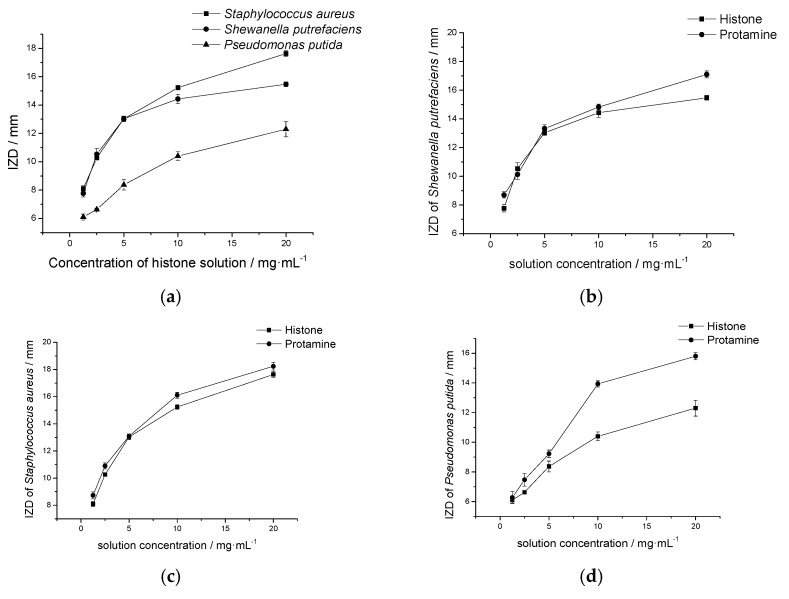
The inhibitory zone diameter (IZD) of histone and protamine against *Shewanella putrefaciens*, *Pseudomonas putida*, and Staphylococcus aureus as the concentration changes. (**a**) The IZD of histone against *Shewanella putrefaciens*, *Pseudomonas putida*, and *Staphylococcus aureus*; (**b**) the comparison of IZD of histone and protamine against *Shewanella putrefaciens*; (**c**) the comparison of IZD of histone and protamine against *Staphylococcus aureus*; (**d**) the comparison of IZD of histone and protamine against *Pseudomonas putida*.

**Figure 5:**
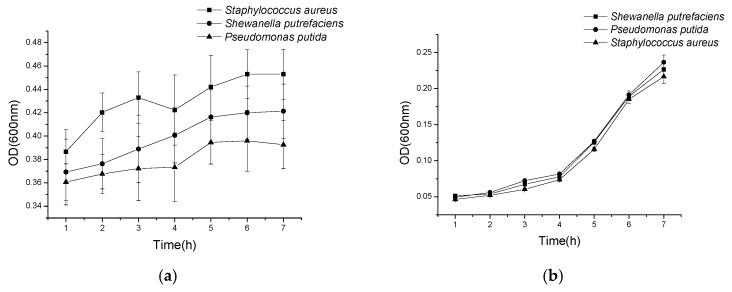
The optical density (OD) value of histone and the blank control group changed with the trend of time. (**a**) The OD value of histone changed with the trend of time; (**b**) the OD value of the blank control group changed with the trend of time.

The bacteriostasis circle method was used to evaluate the antimicrobial activity of histone against *Pseudomonas putida*, *Shewanella putrefaciens*, and *Staphylococcus aureus*, respectively. As shown in Figure 4a, histone showed a significant inhibition to the three bacteria and the inhibition improved with the increase of the concentration of histone. In addition, the antimicrobial effect of histone to *Staphylococcus* was observed to be the best, whereas when the concentration was 10 mg/mL, the inhibitory zone diameter (IZD) of *Staphylococcus aureus*, *Shewanella putrefaciens*, and *Pseudomonas putida* were 15.23, 14.43, and 10.4 mm, respectively (Figure 4a). When compared with protamine at the same concentration, the observed antimicrobial activity of histone to *Staphylococcus aureus* and *Shewanella putrefaciens* was almost the same of that of protamine (Figure 4b, 4c), although it was much lower than that of protamine against *Pseudomonas* (Figure 4d). Meanwhile, the OD method results showed that the optical density (OD) value of bacteria suspension with histone increased much slower (Figure 5a), while the bacteria suspension without histone increased rapidly (Figure 5b).

#### 2.4.2. Antimicrobial Activity of Histone after Modification

**Figure 6:**
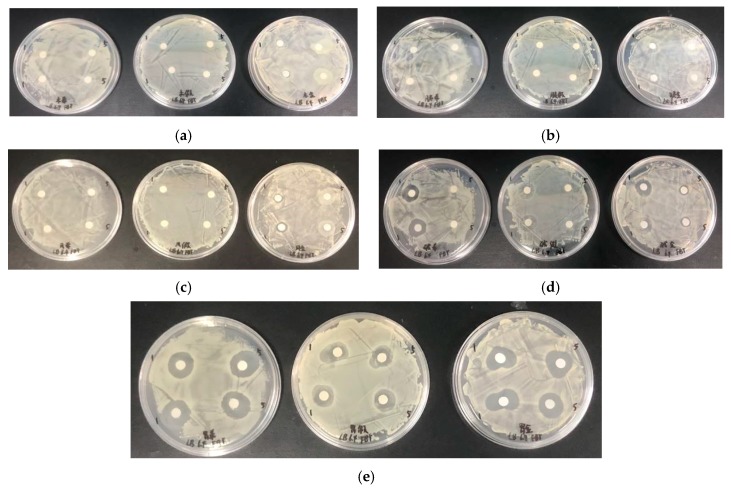
The results of the bacteriostasis activity test of the products of pepsinase hydrolyzed histone at 1 h and 5 h. (**a**) The results of papain enzymatic hydrolysis products; (**b**) the results of trypsin enzymatic hydrolysis products; (**c**) the results of flavourzyme enzymatic hydrolysis products; (**d**) the results of alcalase enzymatic hydrolysis products; (**e**) the results of pepsin enzymatic hydrolysis products.

The antimicrobial activity of histone products after hydrolysis by papain, trypsin, flavor protease, and alkaline protease was investigated. No antimicrobial activity was observed (Figure 6a–c), except that the products hydrolyzed by alkaline protease for 1 h showed slight antimicrobial activity against *Shiva* and *Staphylococcus* (Figure 6d).

**Figure 7:**
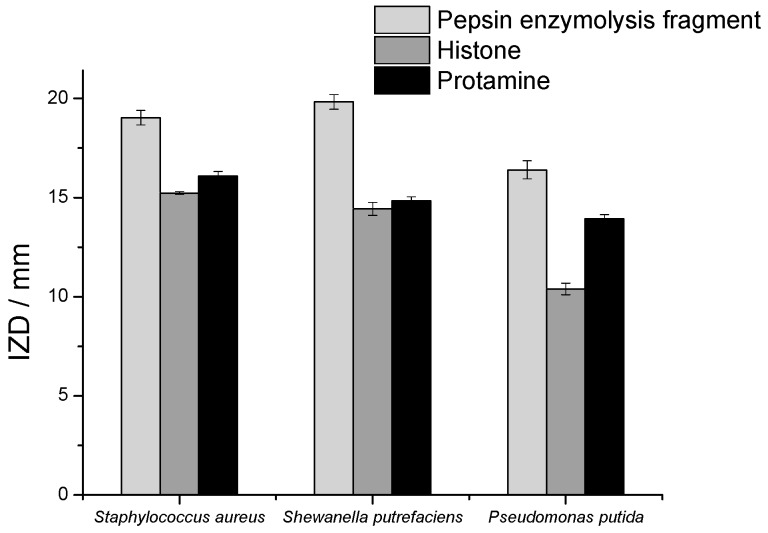
The IZD of pepsin enzymolysis fragment and histone and protamine against *Shewanella putrefaciens*, *Pseudomonas putida*, and *Staphylococcus aureus* at a concentration of 10 mg/mL.

**Figure 8:**
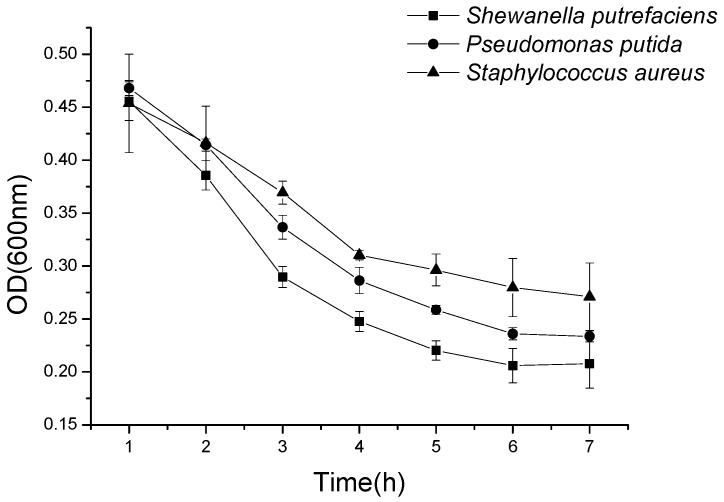
The OD value of the pepsin enzymolysis fragment changed with the trend of time.

The bacteriostasis of the histone products after pepsin hydrolysis at 1 h and 5 h is shown in Figure 6e. Compared with the other four enzymes, the enzymatic fragments of pepsin showed higher bacteriostasis and the fragments of pepsin hydrolyzed for 5 h revealed slightly better bacteriostasis than those of enzymatic hydrolyzed for 1 h, respectively. At the concentration of 10 mg/mL, the 5 h segment of pepsin enzymatic hydrolysis with an antibacterial ring diameter of about 19.03 mm for *Shiva*, 19.83 mm for *Staphylococcus*, and 16.4 mm for *Pseudomonas putida* (Figure 7), which was increased by 31.87%, 30.2%, and 57.7% compared with the diameters of the original histones, which were 14.43 mm, 15.23 mm, and 10.4 mm, respectively. Meanwhile, the OD method results showed that the OD value of bacterial suspension with histone after digestion by pepsin decreased rapidly (Figure 8).

## 3. Discussion

Past studies suggest that histone H1 is known to play a key role in fish immunity. Richards et al. (2001) extracted a 20.7 KDa antibacterial protein from *Atlantic salmon* liver, which was identified as histone H1 by tandem nanoelectrospray mass spectrometry. The minimum inhibitory concentration (MIC) of the protein against *E. coli* D31 was 31 µg/ml [11]. Patrzykat et al. (2001) reported the purification of an antimicrobial peptide (HSDF-1) from the mucus and serum of *coho salmon, Oncorhynchus kisutch*, which was a 26-residue *N*-terminal fragment of histone H1, could inhibit the growth of *A. salmonicida*, *V. anguillarum*, or *Salmonella enterica serovar typhimurium* [20]. On the basis of these results, in this study, histone was extracted from immature gonads of *Atlantic salmon* males that were discarded during commercial fishing operations, and the antibacterial activity was estimated.

In the storage of aquatic products, specific spoilage organisms (SSO) dominate the spoilage of food [21]. *Shewanella putrefaciens* and *Pseudomonas putida* are both common SSO in marine fish, which often appeared in salmon and turbot preserved at low temperatures [22,23]. In addition, *Staphylococcus aureus*, which was a common pathogen in aquatic products, was also selected as the test strain.

Using data analysis software, the peptide mass was extracted from the original documents in order to compare it with the UniProt data bank. The information extracted from the analysis software was uploaded into the MASCOT software, and various parameters such as enzymolysis, chemical modification, and corresponding database were set. On the basis of probability scoring, the software matches the actual detected peptide information with the information in the database and provides reasonable scores and coverage information. In this way, the extracted protein was finally determined as histone rather than protamine, where the observed molecular weight of histone was much bigger than that of protamine.

The enzymolysis method has been often applied in protein modifications, because some bioactive peptides may hide in some of their structures or sequences [24]. According to a previous report, it is possible to obtain peptide segments with high activity by using different enzymes to hydrolyze the proteins [25]. Furthermore, the preparation of antimicrobial peptides by enzymolysis is environmental friendly and easy to control, which is considered the most promising method for mass production of antimicrobial peptides [24]. Therefore, in our study, five common proteases were selected for enzymatic hydrolysis of the extracted histones. Compared with the original histone, the fragments obtained by the pepsin hydrolysate have higher antibacterial activity, which was increased by 31.87%, 30.2%, and 57.7%, while the molecular weight was 15% to 30% lower than that of the original histone. Some other studies have reported that histone proteins or their derivatives have some antibacterial activity, but because of the large molecular weight and functional regions unrelated to antibacterial activity, the complete histones may have some defects when used as intracellular antibacterial factors. Meanwhile, some of the antimicrobial peptides were generated by the degradation of some precursor proteins. Cho et al. (2002) reported that some histone-derived antimicrobial peptides with high similarity to histone amino sequences existed on the body surface and in vivo of fish were mainly derived from histone H1, H2A, and H2B [26]. Nam et al. found that the N-terminal of histone H1 exacted from the testis and ovaries of flouncy (*Paralichthys olivaceus*) was similar to the known antimicrobial peptides, which had strong antibacterial activity to both G^+^ and G^−^ bacteria [27]. The three fragments resultant from the pepsin digestion in this study might be histone-derived antimicrobial peptides, and we will isolate them and analyze their sequence and antimicrobial activity, respectively, in future research.

## 4. Materials and Methods 

### 4.1. Materials

Fresh Atlantic salmon spermary was purchased from Shandong Oriental Ocean sci-tech Co., Ltd, China. Pepsin, trypsin, papain, flavoring protease, and alkaline protease were purchased from Solarbio (Beijing, China). Protamine was purchased from Sigma Company. *Staphylococcus aureus*, *Pseudomonas putida*, and *Shewanella putrefaciens* were obtained from the food safety laboratory of Ocean University of China. All other reagents used in the experiments were chemically pure.

### 4.2. Histone Extraction

One hundred grams of fresh raw Atlantic salmon fish sperm were homogenized for 1 min in 200 mL of NaCl solution (0.14 mol/L). Then, it was stirred in ice bath for 20 min and treated for 10 min, followed by centrifuging at 4000 r/min for 10 min at 4 °C, after which the precipitate was collected. The operation was repeated once and the final precipitation obtained was ribonucleoprotein (DNP). One gram of DNP was mixed in 4 ml 5%(v/v) sulfuric acid, followed by ultrasound extraction at 10 KHz for 20 min, and centrifuged at 4000 r/min for 10 min at 4 °C. The supernatant was collected, and mixed with triple volume of 95% cold ethanol, and then the final precipitate was collected as crude protein.

The crude protein sample was then dried by vacuum freeze-drying and dissolved in ultrapure water to a final concentration of 10 mg/mL. Later, the pH was adjusted to 6–7, and the solution was moved in dialysis bags and dialyzed in ultra-pure water for more than 24 h with an ultra-pure water replacement every 8 h of intervals. Processed samples were collected for SDS-PAGE and Tris-Tricine-SDS-PAGE electrophoresis.

### 4.3. Histone Identification

The histone on the SDS-PAGE gel was identified by LC-MS-MS. Firstly, the clear histone band on the SDS-PAGE gel was cut off and treated with water and decolorizing solution (50% acetonitrile, 25 mM NH_4_HCO_3_), respectively. After vacuuming the acetonitrile, 10 mM DTT was added and incubated in a 56 °C water bath for 1 h. Afterwards, the DTT was removed, 55 mM iodacetamide (C_2_H_4_INO, Iam) was added, and it was incubated in a dark room for 45 min. The colloidal particles were collected and removed Iam, 25 mM NH_4_HCO_3_ was added for cleaning, and decolorizing solution was added for further cleaning. Finally, enzyme storage solution at 1 μg/μL was diluted 15 times with 25 mM NH_4_HCO_3_ and added into the cleaned colloidal particles, followed by addition of 25 mM NH_4_HCO_3_ to soak the colloidal particles, and then digestion at 37 °C overnight. Then, 0.1% FA of final concentration was added to stop digestion, and 10 µL of the sample was drawn for detection with mass spectrometer MicrOTOF-QII (BrukerDaltonics). After detection, the mass spectrogram data was extracted for analysis. Data analysis software was used to mark the peaks, and Mascot search engine version 2.3.01 was then used to search the Uniprot database, respectively.

### 4.4. Histone Modification

Papain, pepsin, trypsin, flavor protease, and alkaline protease were chosen for enzymatic hydrolysis. The enzyme was added at a ratio of 1:30 (g/g protein) to the histone with a concentration of 10 mg/mL. The enzymatic reaction was carried out in a shaker at the optimum condition of the enzyme according to its specification. The products of enzymatic hydrolysis for 1 h and 5 h were selected, respectively. The enzymes were inactivated in boiling water bath for 3–5 min before further use for the bacteriostasis experiment and electrophoresis.

### 4.5. Determination of Bacteriostatic Activity

The bacteriostasis of the histone before and after modification was preliminarily verified by the bacteriostasis circle method and the optical density (OD) method. *Pseudomonas putida*, *Shewanella putrefaciens*, and *Staphylococcus aureus* were chosen as test strains. After culture for 12 h, 100 µL bacterial suspension was evenly coated on the sterilized medium by spreaders, and then the pre-sterilized filter paper disc was dipped in histone solution and placed on the plates, and then incubated at 30 °C for 24 h. Subsequently, the bacteriostatic circles were successfully observed. Protamine was used as the positive control and sterile water was used as the negative control. The OD value method was used to detect the change in the optical density curves to time by mixing 50 μL bacterial suspension with 50 μL histone solution and cultured for six hours.

## 5. Conclusions

In this study, histone was extracted from immature testis of *Atlantic salmon* by acid extraction, modified by proteinases, and its antibacterial activity before and after modification was investigated. The results showed that the extracted histone with a molecular weight of about 30 KDa showed broad-spectrum antibacterial activity. Whereas, after modification with pepsin, histone was hydrolyzed into three smaller fragments, and the antibacterial activity against *Staphylococcus aureus*, *Shewanella putrefaciens*, and *Pseudomonas putida* was found to be increased by 31.87%, 30.2%, and 57.7%, respectively. All the results obtained in this study emphasize and conclude that histone could be used as a potential preservative in the preservation application of various seafood in the food industry.

## Figures and Tables

**Table 1:** The search conditions and parameters of histone.

Parameter Options	Parameter Determination
Type of search	MS/MS Ion Search
Enzyme	Trypsin
Fixed modifications	Carboxymethyl (C)
Variable modifications	Gln- Epyro-Glu (N-term Q), Oxidation (M)
Mass values	Mono isotopic
Protein mass	Unrestricted
Peptide mass tolerance	−/+ 15 ppm
Fragment mass tolerance	−/+ 20 mmu
Max missed cleavages	1
Instrument type	Default
Number of queries	10,497
Database	uniprot20180305 (556,825 sequences; 199,652,254 residues)
Taxonomy	Metazoa (Animals) (101,714 sequences)

**Table 2:** The partial comparison results in the Uniprot database.

Accession	Description	Mass	Score	Matches	Sequences	Coverage
P06350	Histone H1 OS = Oncorhynchus mykiss OX = 8022 PE = 1 SV = 2	20791	1268	84(52)	12(12)	38%
P04264	Keratin, type II cytoskeletal 1 OS = Homo sapiens OX = 9606 GN = KRT1 PE = 1 SV = 6	66173	722	36(23)	17(15)	25%
P13645	Keratin, type I cytoskeletal 10 OS = Homo sapiens OX = 9606 GN = KRT10 PE = 1 SV = 6	59024	366	27(13)	17(10)	24%
P35908	Keratin, type II cytoskeletal 2 epidermal OS = Homo sapiens OX = 9606 GN = KRT2 PE = 1 SV = 2	65683	329	20(12)	14(10)	23%
P35527	Keratin, type I cytoskeletal 9 OS = Homo sapiens OX = 9606 GN = KRT9 PE = 1 SV = 3	62259	159	15(7)	10(7)	17%
P42212	Green fluorescent protein OS = Aequorea victoria OX = 6100 GN = GFP PE = 1 SV = 1	26985	143	11(6)	6(3)	27%
P02754	Beta-lactoglobulin OS = Bos taurus OX=9913 GN = LGB PE = 1 SV = 3	20276	122	6(4)	4(3)	17%
P02533	Keratin, type I cytoskeletal 14 OS = Homo sapiens OX = 9606 GN = KRT14 PE = 1 SV = 4	51877	119	8(4)	7(4)	16%
